# Effect of Papaverine on Left Internal Mammary Artery Flow: Topical Spraying *versus* Perivascular Injection Method

**DOI:** 10.21470/1678-9741-2019-0126

**Published:** 2020

**Authors:** Girish Gowda S L, Jayanth Kumar H V, Veeresh G S, Anand Kuriyan Mathew, Manjunath Cholenahally Nanjappa

**Affiliations:** 1 Department of Cardiothoracic and Vascular Surgery, Sri Jayadeva Institute of Cardiovascular Sciences and Research, Bangalore, Karnataka, India.

**Keywords:** Papaverine, Mammary Artery, Biometry, Infections, Vasodilator Agents

## Abstract

**Objective:**

To analyze two techniques of papaverine application, topical spray on the harvested left internal mammary artery (LIMA) and perivascular injection, to find out their ability to improve LIMA flow.

**Methods:**

Forty patients were randomized into two groups. In Group 1, papaverine was sprayed on the harvested pedunculated LIMA. In Group 2, papaverine was delivered into the perivascular plane. Drug dosage was the same for both groups. LIMA flow was measured 20 minutes after applying papaverine. Blood flow was recorded for 20 seconds and flow per minute was calculated. The systemic mean pressures were maintained at 70 mmHg during blood collection. The data collected was statistically evaluated and interpreted.

**Results:**

The LIMA blood flow before papaverine application in the Group 1 was 51.9±13.40 ml/min and in Group 2 it was 55.1±15.70 ml/min. Statistically, LIMA flows were identical in both groups before papaverine application. The LIMA blood flow, post papaverine application, in Group 1 was 87.20±13.46 ml/min and in Group 2 it was 104.7±20.19 ml/min. The Group 2 flows were statistically higher than Group 1 flows.

**Conclusion:**

Papaverine delivery to LIMA by the perivascular injection method provided statistically significant higher flows when compared to the topical spray method. Hence, the perivascular delivery of papaverine is more efficient than the spray method in improving LIMA blood flow.

**Table:** 

Abbreviations, acronyms & symbols	
CABG	= Coronary artery bypass grafting
IV	= Intravenous
LIMA	= Left internal mammary artery
NTG	= Sodium nitroprusside

## INTRODUCTION

The left internal mammary artery (LIMA) graft is the most commonly used arterial graft in coronary artery bypass grafting (CABG)^[1]^. LIMA spasm is an undesirable complication during LIMA harvesting. LIMA spasm can lead to immediate and late morbidity and mortality of the patient after CABG^[2]^. Various drugs and precautions are used during LIMA harvesting to prevent spasm. Many studies have shown papaverine to increase blood flow compared to control^[3-6]^, and sodium nitroprusside (NTG) has also been used in few studies and found to be effective in relieving LIMA spasm and improving LIMA flow^[7,8]^. Intraluminal instillation of papaverine has shown to cause mechanical injury to the lumen of the mammary artery, causing dissections and medial disruption^[9,10]^. Hence, perivascular application of papaverine is the safest method of papaverine delivery. We use a combination of papaverine and NTG to prevent LIMA spasm and to improve the flow. Papaverine can be applied perivascularly by two methods: topical spray and perivascular injection. We have evaluated these two techniques to find out their ability to improve the LIMA flow.

## METHODS

**Fig. 1:**
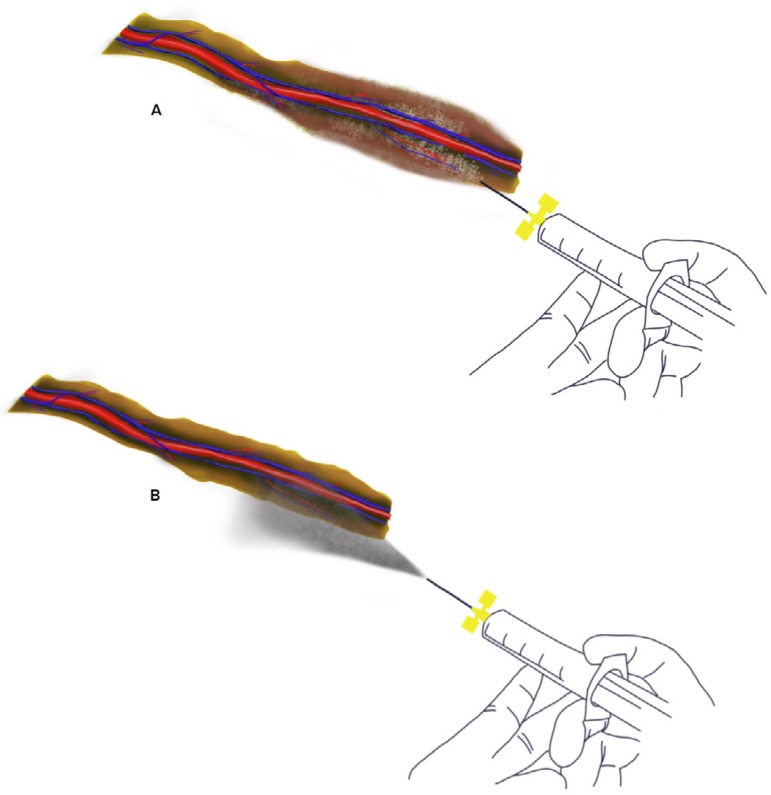
Diagrammatic representation of two different methods of papaverine delivery adopted in this study. A) Perivascular method; B) spray method.

Forty patients who underwent CABG were randomized into two groups. The preoperative risk factors and patients’ characteristics were statistically the same for both groups. Midline sternotomy was done in all patients followed by LIMA harvesting with a wide pedicle up to bifurcation. LIMA was harvested by a single surgeon with low power electrocautery (30 W) in fulgurate mode. Major branches were controlled with hemoclips. Systemic heparinization was done with 400 units/kg heparin dose and LIMA was transected three minutes after the heparin dose administration. LIMA blood flow was collected for 20 seconds and flow per minute was calculated. After measuring the blood flow, LIMA was clipped at the distal end. Papaverine solution was prepared by adding 30 mg papaverine in 100 ml of normal saline making it 0.3 mg/ml. In Group 1, papaverine was sprayed topically from a distance of 5 to 10 centimeters to the harvested pedunculated LIMA using a blunt tipped intravenous (IV) cannula (Vasofix Centro 24G) (Figure 1B). In Group 2, papaverine was delivered by perivascular injection using a blunt tipped IV cannula (Vasofix Centro 24G) (Figure 1A), taking precaution not to injure LIMA. Drug dosage was the same for both groups. Figure 1 shows a diagrammatic representation of the two different methods of papaverine delivery adopted in this study. The harvested LIMA was wrapped with a gauze soaked in the solution and was placed in the left hemithorax for 20 minutes. LIMA flow was measured 20 minutes after applying papaverine. Blood flow was recorded for 20 seconds and flow per minute was calculated. The systemic mean pressures were maintained at 70 mmHg during blood collection. The data collected was statistically evaluated and interpreted.

### Statistical Analysis

Continuous variables are reported as mean ± standard deviation and were compared using the *t*-test for normal distributions. The *t*-test for one mean and comparison of the mean was done. A pilot study was done, and the sample size was calculated. Student’s paired *t*-test and unpaired *t*-test were done, and reported *P-*values of < 0.05 were considered statistically significant. Statistical analysis was performed with MedCalc statistical software.

## RESULTS

**Fig. 2:**
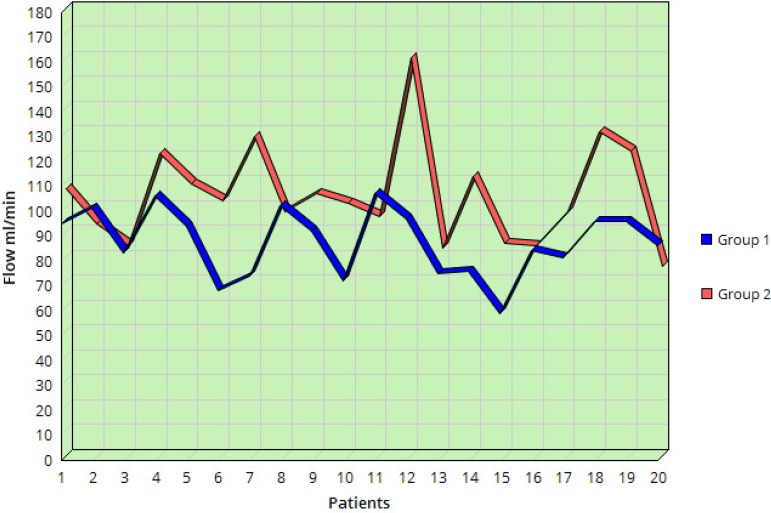
Graph depicting the distribution of flow in Group 1 and Group 2 after papaverine application.

The LIMA blood flow before papaverine application in Group 1 was 51.9±13.40 ml/min and in Group 2 it was 55.1±15.70 ml/ min. Statistically, LIMA flows were identical in both groups before papaverine application. The LIMA blood flow after papaverine application in Group 1 was 87.20±13.46 ml/min and in Group 2 it was 104.7±20.19 ml/min (Table 1). The unpaired *t*-test showed that Group 2 flow was statistically higher than Group 1 flow (*P*<0.005). Figure 2 demonstrates the graph depicting the distribution of flow in Groups 1 and 2 after papaverine application.

**Table 1:** Left internal mammary artery flow measurements and their statistical analysis.

	Pre-papaverine application flow (ml/min)	Post-papaverine application flow (ml/min)	Student's paired *t*-test
Group 1	51.9±13.40	87.20±13.46	< 0.05 (significant)
Group 2	55.1±15.70	104.7±20.19	< 0.05 (significant)
Student's unpaired *t*-test	0.4923 (not significant)	< 0.05 (significant)	

## DISCUSSION

The LIMA to left anterior descending artery anastomosis is the most commonly performed bypass grafting. LIMA is an important and a vital graft due to its inherent ability to last for long time when compared to other graft conducts. LIMA spasm is a well-recognized complication encountered after LIMA harvesting during CABG. We have used various vasodilators to either prevent or treat LIMA spasm, but the best agent is not known yet^[11-13]^. Clinically, papaverine is the most commonly studied and used vasodilator drug. Papaverine can be delivered by various ways, like injection into endothoracic fascia before LIMA dissection, pedicled perivascular injection, topical spray, and intraluminal administration with or without hydrostatic dilation. Few studies have shown that the intraluminal administration type of delivery improves LIMA flow very drastically^[14]^, but it is associated with mechanical injury to the lumen of the mammary artery causing dissections and medial disruption^[9,10]^. A study done by Hausmann^[13]^ reported no difference in LIMA flow when papaverine was delivered intraluminally or injected in the pedicle after LIMA dissection. Papaverine injection into endothoracic fascia before LIMA dissection is associated with few complications, like damage to the vessel wall, making it not usable. Perivascular injection and topical spray are the methods with very minimal chances of injuring LIMA. The perivascular injection delivery method allows better availability of papaverine to the LIMA than the topical spray method. This injection method helps in achieving prolonged exposure of papaverine to LIMA. Transit time flow measurement is a most valuable and accurate method of measuring the blood flow through the grafts^[13]^. In our study, LIMA flow in Group 1 was statistically different from Group 2. Papaverine dosage used in the literature has varied from 0.3 mg/mL to 1.5 mg/mL and to a maximum of 50 mg^[14]^. We used papaverine with a 0.3 mg/ml concentration. Papaverine delivery to the LIMA pedicle after LIMA dissection definitely improves the flow volume. We observed that administering papaverine by perivascular injection method helps in achieving high flows when compared to the spray method.

## CONCLUSION

Papaverine delivery to LIMA by perivascular injection method provided statistically significant higher flow when compared to the topical spray method. Hence, perivascular delivery of papaverine is more efficient than the spray method in improving LIMA blood flow.

**Table:** 

Authors' roles & responsibilities	
GGSL	Substantial contributions to the conception or design of the work; or the acquisition, analysis, or interpretation of data for the work; drafting the work or revising it critically for important intellectual content; agreement to be accountable for all aspects of the work in ensuring that questions related to the accuracy or integrity of any part of the work are appropriately investigated and resolved; final approval of the version to be published
JKHV	Agreement to be accountable for all aspects of the work in ensuring that questions related to the accuracy or integrity of any part of the work are appropriately investigated and resolved; final approval of the version to be published
VGS	Drafting the work or revising it critically for important intellectual content; agreement to be accountable for all aspects of the work in ensuring that questions related to the accuracy or integrity of any part of the work are appropriately investigated and resolved; final approval of the version to be published
AKM	Agreement to be accountable for all aspects of the work in ensuring that questions related to the accuracy or integrity of any part of the work are appropriately investigated and resolved; final approval of the version to be published
MCN	Final approval of the version to be published
